# Electroencephalographic features in pediatric patients with moyamoya disease in China

**DOI:** 10.1186/s41016-019-0179-2

**Published:** 2020-01-13

**Authors:** Jia Lu, Qing Xia, Tuanfeng Yang, Jun Qiang, Xianzeng Liu, Xun Ye, Rong Wang

**Affiliations:** 1 Department of Neurology, Peking University International Hospital, No.1 Life Park Road, Changping District, Beijing, 102206 China; 2 Department of Neurosurgery, Peking University International Hospital, Beijing, 102206 China; 3 Department of Neurosurgery, Beijing Tiantan Hospital, Beijing, 100070 China

**Keywords:** Electroencephalography, Moyamoya disease, pediatric, seizure, epilepsy

## Abstract

**Background:**

Moyamoya disease (MMD) is a relatively important and common disease, especially in East Asian children. There are few reports about EEG in children with MMD in China till now. This study is aimed to analyze the electroencephalographic features of MMD in pediatric patients in China preliminarily.

**Methods:**

Pediatric patients with MMD who were hospitalized in Peking University International Hospital and Beijing Tiantan Hospital from January 2016 to December 2018 were collected. Clinical and electroencephalography (EEG) findings were analyzed retrospectively.

**Results:**

A total of 110 pediatric patients with MMD were involved, and 17 (15.5%) cases had a history of seizure or epilepsy. Ischemic stroke was associated with a 1.62-fold relative risk of seizure. A subset of 15 patients with complete EEG data was identified. Indications for EEG in patients with MMD included limb shaking, unilateral weakness, or generalized convulsion. Abnormal EEG was seen in 14 (93.3%) cases, with the most common findings being focal slowing 12 (80.0%), followed by epileptiform discharge 10 (66.7%), and diffuse slowing 9 (60.0%). “Re-build up” phenomenon on EEG was observed in one patient.

**Conclusions:**

Seizure and abnormal background activity or epileptiform discharge on EEG were common in pediatric patients with MMD. EEG may play a role in differential diagnosis among the transient neurological events in MMD such as transient ischemic attack and seizure.

## Background

Moyamoya disease (MMD) is a chronic, occlusive cerebrovascular disease with an uncertain etiology characterized by bilateral steno-occlusive changes at the terminal portion of the internal carotid artery and an abnormal vascular network at the base of the brain [1–3]. It is a relatively important and common disease, especially in East Asian children [1–3]. Clinical experience and basic knowledge about MMD have increased considerably since 1969 when Suzuki and Takaku firstly reported this disease [4].

In view of the abnormal pathology of this progressive cerebral vasculopathy, it is not difficult to understand the recurrent and stereotyped symptoms in MMD, such as transient ischemic attack (TIA), ischemic, or hemorrhagic stroke [1–3]. Seizure is another prominent transient event in MMD, with a prevalence of 11–18% in pediatric population [5, 6] and 18–24% in adults [7, 8]. Therefore, diagnostic and therapeutic dilemma arises from these transient neurological events.

Electroencephalography (EEG) is not commonly used as a diagnostic tool in MMD. However, it can play a relatively role in the differential diagnosis of TIA-like symptoms [9, 10]. Up to now, there are few reports about EEG in children with MMD in China.

## Methods

### Subjects

This was a retrospective study of MMD. The inclusion criteria included children aged up to 18 years old who were diagnosed with MMD by digital subtraction angiography (DSA) or magnetic resonance angiography (MRA). The clinical diagnosis of MMD was based on the guidelines of the Research Committee on MMD (spontaneous occlusion of the DSA circle of Willis) of Japan in 2012 [11], and moyamoya syndrome was excluded. The clinical information and EEG data for patients with MMD in Peking University International Hospital and Beijing Tiantan Hospital from January 2016 to December 2018 were required.

For each patient, the following information was collected: gender, age, age at neurological event onset, primary symptoms, seizure history, surgical history, personal and family history, and results of comprehensive general and neurological examinations, such as computed tomography (CT), magnetic resonance imaging (MRI), MRA, SPECT (single photon emission computed tomography), CT perfusion (CTP), and DSA.

Follow-up was conducted by either face-to-face or telephonic interview. The last follow-up was conducted in March 2019. The prognosis of seizure was determined according to Engel’s classification [12]. Class I: completely seizure-free, auras only, atypical early postoperative seizures, or generalized convulsions only with anti-epileptic drug discontinuation; Class II: 90% or greater seizure reduction or nocturnal seizures only; Class III: 75%–90% seizure reduction; Class IV: < 75% seizure reduction, no significant change, or worse seizures.

### EEG

The scalp video EEG (VEEG) recording was finished according to the international 10-20 system (Nicolet, USA). The reference point was put at midline just 1 cm anterior to Cz. Electrocardiogram was recorded with two electrodes placed at bilateral midpoint of clavicle, respectively. Electrode impedance was lower than 5 kilohm. VEEG was recorded continuously for more than 12 h.

For patients with complete EEG data, indications for EEG and detailed seizure history were summarized. Two different neurologists who were blinded with patients’ clinical information interpreted the EEG recordings.

The following characteristics and phrases of the EEG database were evaluated: background activity abnormalities, focal slowing, diffuse slowing, epileptiform discharge, amplitude asymmetry, breach rhythm, and rhythmic delta activity.

### Statistical analysis

Descriptive summaries were reported as the mean ± standard deviation for continuous variables and as frequency (percentage) for categorical variables.

## Results

A total of 110 pediatric patients with MMD were involved (Table 1). Seventeen (15.5%) cases had a history of seizure or epilepsy. Among the 17 patients, 15 cases received revascularization and 14 cases were Engel class I, and only 1 patient was Engel class III after operation.

**Table 1:** Demographics of patients with MMD

Variables	Description
*n*	110
Male/female	58/52
Age at onset of neurological event (years)	9.9 ± 4.0
Seizure or epilepsy (*n*)	17
Hemispheres with history of surgery (*n*)	158
Ischemic stroke (*n*)	39
Hemorrhagic stroke (*n*)	4

Among 39 of 110 patients with a diagnosis of ischemic stroke, 8 of 39 (20.5%) cases had a history of seizure or epilepsy. However, among 71 of 110 without a diagnosis of ischemic stroke, 9 of 71 (12.7%) cases had seizure or epilepsy. The relative risk of seizure for ischemic stroke was 1.62. Four patients had a diagnosis of hemorrhagic stroke, but no seizure occurred in the subset.

Among the 110 patients with MMD, a cohort of 15 patients with complete EEG data was indentified (Table 2). There were 7 males and 8 females with a mean age of 9.1 ± 4.1 years. Six (40.0%) cases had a history of seizure or epilepsy. Seven patients had evidence of ischemic stroke on MRI. No patient had a history of hemorrhagic stroke. All the 15 patients received revascularization in 26 hemispheres, and encephaloduroarteriosynangiosis (EDAS) was the first choice for pediatric patients.

**Table 2:** Demographics of patients with EEG

Number	Gender	Age	Symptom	Ischemic stroke on MRI	SPECT hypoperfusion	CTP hypoperfusion	Revascularization	Engel’s classification
1	*M*	8	Cerebral infarction	Both FP	Both FPT	Not done	EDAS; MBH	-
2	*M*	11	TIA	Right FPO, left O	Both H	Both H	MBH bilaterally	-
3	*M*	5	TIA, SGTCS	Right PO	Both H	Not done	EDAS bilaterally	I
4	*F*	9	TIA	-	Not done	Both FP	Bypass bilaterally	-
5	*F*	7	TIA	-	Not done	Left FT	EDAS unilaterally	-
6	*F*	14	TIA	-	Not done	Both FPT	Bypass bilaterally	-
7	*F*	12	TIA, SGTCS	-	Not done	Both FPT	Bypass+EDAS unilaterally	I
8	*M*	6	Headache, TIA	-	Right basal ganglia, right thalamus	Both FP, right T	EDAS bilaterally	-
9	*F*	5	TIA	-	Right F	Not done	EDAS bilaterally	-
10	*M*	17	SGTCS, SPS	Left P	Left TP, both O	Not done	Bypass+EDAS unilaterally	I
11	*F*	4	TIA	Left FP	Left FPT	Not done	EDAS bilaterally	-
12	*M*	7	TIA, SGTCS	Both F	Both FP, left basal ganglia	Both F	Bypass bilaterally	I
13	*M*	7	TIA	-	Not done	Left FPO	Bypass+EDAS unilaterally	-
14	*F*	15	TIA, SGTCS	-	Not done	Both F	Bypass+EDAS; bypass	I
15	*F*	9	TIA, SGTCS	Both F	Not done	Both FP, right T	Bypass+EDAS; bypass	I

*M*, male; *F*, female; TIA, transient ischemic attack; *SGTCS*, secondarily generalized tonic-clonic seizure; *SPS*, simple partial seizure; *SPECT*, single photon emission computed tomography; *CTP*, computed tomography perfusion; *F*, frontal; *P*, parietal; *T*, temporal; *O*, occipital; *EDAS*, encephaloduroarteriosynangiosis; *MBH*, multiple burr holes

Indications for EEG in patients with MMD were summarized in Table 3. EEG findings in patients with MMD were summarized in Table 4. Abnormal EEG was seen in 14 (93.3%) cases, with the most common findings being focal slowing 12 (80.0%), followed by epileptiform discharge 10 (66.7%), and diffuse slowing 9 (60.0%). Among the 7 patients with evidence of ischemic stroke on MRI, all cases showed corresponding cerebral hypoperfusion on SPECT or CTP, and fixed regional slow wave or epileptiform discharge was identified on EEG.

**Table 3:** Indications for EEG in patients with MMD

Indications	Number
Limb shaking	8
Unilateral weakness	8
Generalized convulsion	6
Dysarthria	3
Numbness	3
Aphasia	2
Memory loss	1
Vision decline	1
Altered mental status	1

**Table 4:** EEG findings in patients with MMD

Findings	Number (frequency)
Any abnormality	14 (93.3%)
Focal slowing	12 (80.0%)
Epileptiform discharge	10 (66.7%)
Diffuse slowing	9 (60.0%)
Asymmetric posterior alpha rhythm	6 (40.0%)
Breach rhythm	4 (26.7%)
Rhythmic delta activity	1 (6.7%)

**Fig. 1:**
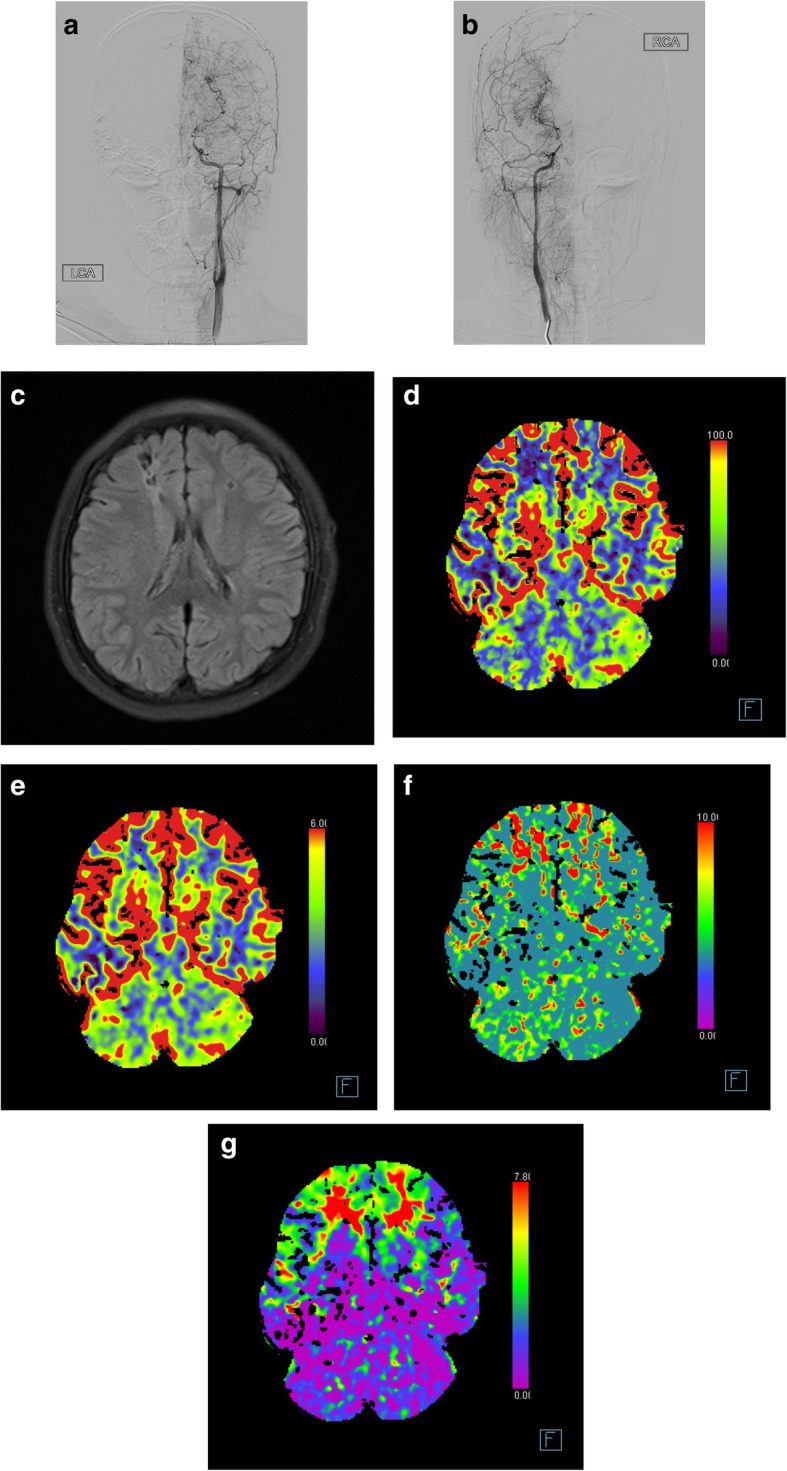
Clinical data of a 9-year-old girl. **a** and **b** Bilateral severe stenosis of terminal internal carotid artery on DSA (**a** left; **b** right). **c** Infarction in both frontal lobes on MRI. **d**–**g** Hypoperfusion in both frontal lobes on CTP. Reduced cerebral blood flow (**d**), relatively normal cerebral blood volume (**e**), increased mean transit time (**f**), and increased Tmax in both frontal lobes (**g**)

The patient number 15 was present in Fig. 1 as an illustrated case. The 9-year-old girl had repeated motor TIA and generalized tonic-clonic seizure (GTCS), with frontal infarction on MRI and hypoperfusion on CTP at admission. Paroxysmal sharp-and-slow waves were recorded diffusely, especially in bilateral frontal areas. After receiving bilateral revascularization 6 months later, the clinical symptoms disappeared and the abnormal EEG activity decreased. A total of 372 spike and sharp waves were recorded during 14-hour preoperative EEG monitoring using its own software packed in Nicolet system, but meanwhile, 181 spike and sharp waves were recorded in the same amount of time after operation.

**Fig. 2:**
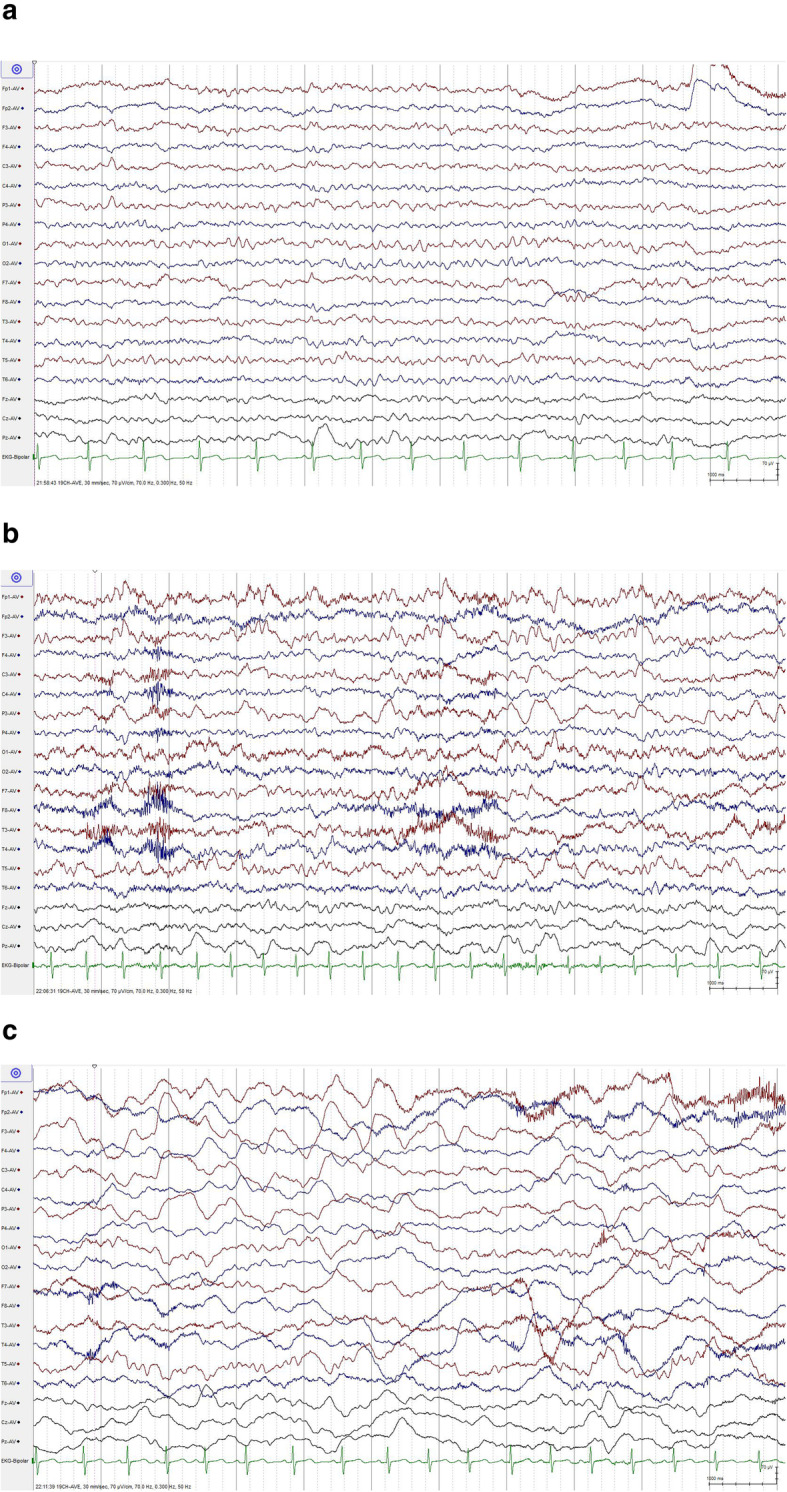
EEG findings before and after hyperventilation in a 10-year-old boy. **a** Before hyperventilation. **b** At the end of hyperventilation. **c** 5 min after hyperventilation. Slow activity was recorded in the left hemisphere during hyperventilation. The slow activity was enlarged and the “re-build up” phenomenon was observed over bilateral hemispheres after hyperventilation

Only one patient finished the hyperventilation, and the “re-build up” phenomenon was observed (Fig. 2).

## Discussion

The two cardinal clinical signs of MMD are ischemic and hemorrhagic attacks. In our cohort, 39 pediatric patients presented with ischemic stroke. In contrast, hemorrhagic stroke only occurred in 4 cases. Epidemiologic study indicated that ischemic stroke was common in MMD for either children or adults in general, whereas hemorrhagic stroke was prominent in adults [1, 2].

Seizure or epilepsy is another common manifestation of MMD [1, 2]. In our study, the prevalence of a seizure history was 15.5% in pediatric patients with MMD, which was similar to the previous reports [5, 6].

In the present study, we found that there existed a very close correlation between ischemic stroke and seizure. Ischemic stroke generated a 1.62-fold risk of seizure compared with those without an ischemic stroke. In adults with MMD, Frechette et al. identified that ischemic stroke was associated with a 2.1-fold relative risk of seizure [8]. Epilepsy in MMD is one of the recognized types of post-stroke epilepsy. The occlusion of middle cerebral artery in MMD is usually accompanied with high NIHSS scores and cortical involvement, both of which are the identified risk factors of post-stroke epilepsy [13–15]. In addition, children are likely to develop into post-stroke epilepsy compared with adults [16, 17].

In pediatric patients with MMD in particular, TIA is sometimes difficult to differentiate from epileptic seizure [9, 10]. In our study, limb shaking and unilateral weakness were the most two common indications for EEG, which demonstrated the dilemma of differential diagnosis between seizure and TIA.

Limb shaking is a common feature in MMD. Kim proposed that limb shaking in MMD may result from a transient hypoperfusion of the contralateral frontoparietal cortex rather than basal ganglia [18]. As for differential diagnosis, myoclonic epilepsy or Jackson’s seizure should be taken into consideration. Epileptiform discharges such as poly-spike waves and dynamic evolution on EEG are likely to point to seizure other than TIA. Therefore, EEG may provide valuable information for differential diagnosis.

Kodama firstly described electroencephalographic findings in children with MMD in 1979, characteristic findings consisting of posterior slow activity, centrotemporal slow activity, re-build up after the end of hyperventilation, and sleep spindle depression [19]. In our study, localized or generalized slow waves and asymmetric posterior alpha rhythms were common features of abnormal background activity. Epileptiform discharge was present in 10 (66.7%) cases. Frechette et al. found that 90% of EEGs were abnormal in adults with MMD, with commonly focal (78%), diffusely slow (68%), or epileptiform discharge (24%) [8].

Research shows that slower waves are generated by the thalamus and by cells in layers II–VI of the cortex. Faster waves derive from cells in layers IV and V of the cortex. All the brain electrical activities are modulated by the reticular activating system [20, 21]. Pyramidal neurons in layers III, V, and VI are exquisitely sensitive to conditions of low oxygen, such as ischemia or hypoperfusion [22].

EEG is a well-established tool for detecting, describing, and monitoring brain function, with sensitivity to changes in the metabolic and electrical activity of neurons that occurs when cerebral blood flow (CBF) is compromised. When normal CBF declines to approximately 25–35 ml/100 g/min, the EEG firstly loses faster frequencies; then, as the CBF decreases to approximately 17–18 ml/100 g/min, slower frequencies gradually increase. This represents a crucial ischemic threshold at which neurons begin to lose their transmembrane gradients, leading to cell death [23, 24]. Hypoperfusion in bilateral hemispheres is common in MMD, thus leading to the abnormal slow activity on EEG.

In our study, all the 7 patients with evidence of ischemic stroke on MRI showed fixed regional slow wave or epileptiform discharge; it was thought that the slow wave and epileptiform discharge in MMD probably reflect secondary cerebral parenchymal injury resulting from hypoperfusion or infarction rather than the characteristics of the disease itself [25].

A “re-build up” phenomenon is the reappearance of polymorphous slow waves several minutes after cessation of the hyperventilation [26]. The mechanism which is responsible for the EEG re-build up phenomenon is still unclear, but several studies have suggested a possible mechanism. Electrophysiological build-up during hyperventilation is based on a reduction of the arterial partial pressure of carbon dioxide as a result of hyperventilation and the consequent reduction of cerebral perfusion [27–29]. The concentration of oxygenated hemoglobin progressively increases during hyperventilation and subsequently decreases after hyperventilation for 5–7 min. The re-build up phenomenon occurs when oxygenated hemoglobin decreases and the deoxygenated hemoglobin increases [30]. Regional cerebral blood flow decreases after hyperventilation in a region where cerebrovascular reactivity is disturbed. Therefore, the regional cerebral hypoxia and the disturbance in oxygen metabolism play important roles in the occurrence of the re-build up phenomenon after hyperventilation. “Re-build up” is the manifestation not only of ischemic hypoxia but also of the hypoxic hypoxia seen characteristically in MMD [27, 31–33].

Hyperventilation produces vasoconstriction and therefore risk in a vascularly impaired patient, so some researchers regard MMD as a contraindication for hyperventilation during routine EEG recording. Therefore, we did not perform hyperventilation as a routine test and only a 10-year-old boy finished hyperventilation under careful monitoring. Fortunately, significant complications including motor TIA and stroke were not observed during and after hyperventilation.

Some researchers suggested that hyperventilation during EEG recording in MMD is a relatively safe and valuable test [25, 34]. When a patient presented with characteristic EEG changes after hyperventilation, MMD was considered as a probable etiology even without MRA or DSA. Researches also emphasized prognostic value of the rebuild-up phenomenon in pediatric population [25, 34]. However, the hyperventilation should be induced under close and careful supervision of an experienced examiner and should be stopped immediately when any clinical symptoms attack.

Revascularization is the primary therapeutic method for MMD and there is no sufficient evidence for drug therapy. In our study, 14 cases showed favorable seizure control among the 15 patients who received revascularization with a history of seizure. In the illustrated case, the abnormal EEG activity decreased after operation following with clinical remission. Previous studies described the similar clinical prognosis along with post-operation EEG improvement [35, 36]. Therefore, EEG may provide additional prognostic information in MMD after surgery.

Furthermore, it is important to follow patients for possible recurrence of symptoms associated with MMD after operation. Ma et al. reported the duration of epilepsy as an independent risk factor for recurrent seizure after revascularization in pediatric patients with MMD [6].

## Conclusion

The main findings of this review are that a history of seizure and abnormal background activity or epileptiform discharge on EEG is common in pediatric patients with MMD. Transient neurological events in MMD could result from not only TIA but also seizure, and EEG may play a role in differential diagnosis. The limitations of this study included the fact that it was a retrospective study and involved a relatively small number of subjects. EEG was ordered for patients who had a clinical event suspicious for seizure, so selection bias limited interpretation. Further studies with larger and multicenter samples and long-term follow-up are essential for further research.

## Data Availability

Not applicable

## Abbreviations

CBF: Cerebral blood flow
CT: Computed tomography
CTP: Computed tomography perfusion
DSA: Digital subtraction angiography
EDAS: Encephaloduroarteriosynangiosis
EEG: Electroencephalography
GTCS: Generalized tonic-clonic seizure
MBH: Multiple burr holes
MMD: Moyamoya disease
MRA: Magnetic resonance angiography
MRI: Magnetic resonance imaging
SPECT: Single photon emission computed tomography
SPS: Simple partial seizure
TIA: Transient ischemic attack

## References

[1] Kim JS (2016). Moyamoya disease: epidemiology, clinical features, and diagnosis. J Stroke..

[2] Fujimura M, Bang OY, Kim JS (2016). Moyamoya disease. Front Neurol Neurosci..

[3] Huang S, Guo ZN, Shi M (2017). Etiology and pathogenesis of moyamoya disease: an update on disease prevalence. Int J Stroke..

[4] Suzuki J, Takaku A (1969). Cerebrovascular “moyamoya” disease. Disease showing abnormal net-like vessels in base of brain. Arch Neurol..

[5] Nakase H, Ohnishi H, Touho H (1993). Long-term follow-up study of “epileptic type” moyamoya disease in children. Neurol Med Chir (Tokyo)..

[6] Ma Y, Zhao M, Zhang Q (2018). Risk factors for epilepsy recurrence after revascularization in pediatric patients with moyamoya disease. J Stroke Cerebrovasc Dis..

[7] Jin SC, Oh CW, Kwon OK (2011). Epilepsy after bypass surgery in adult moyamoya disease. Neurosurgery..

[8] Frechette ES, Bell-Stephens TE, Steinberg GK (2015). Electroencephalographic features of moyamoya in adults. Clin Neurophysiol..

[9] Ali S, Khan MA, Khealani B (2006). Limb-shaking transient ischemic attacks: case report and review of literature. BMC Neurol..

[10] Kraemer M, Diehl RR, Diesner F (2012). Differential diagnosis between cerebral ischemia, focal seizures and limb shaking TIAs in moyamoya disease. Br J Neurosurg..

[11] Research Committee on the Pathology and Treatment of Spontaneous Occlusion of the Circle of Willis; Health Labour Sciences Research Grant for Research on Measures for Infractable Diseases (2012). Guidelines for diagnosis and treatment of moyamoya disease (spontaneous occlusion of the circle of Willis). Neurol Med Chir (Tokyo).

[12] Engel J, Van Ness PC, Rasmussen TB, Engel J (1993). Outcome with respect to epileptic seizures. Surgical treatment of the epilepsies.

[13] Rodríguez Lucci F, Alet M, Ameriso SF (2018). Post-stroke epilepsy. Medicina (B Aires)..

[14] Benninger F, Holtkamp M (2017). Epileptic seizures and epilepsy after a stroke: incidence, prevention and treatment. Nervenarzt..

[15] Mikami T, Ochi S, Houkin K (2015). Predictive factors for epilepsy in moyamoya disease. J Stroke Cerebrovasc Dis..

[16] Breitweg I, Stülpnagel CV, Pieper T (2017). Early seizures predict the development of epilepsy in children and adolescents with stroke. Eur J Paediatr Neurol..

[17] Fox CK, Glass HC, Sidney S (2013). Acute seizures predict epilepsy after childhood stroke. Ann Neurol..

[18] Kim HY, Chung CS, Lee J (2003). Hyperventilation-induced limb shaking TIA in moyamoya disease. Neurology..

[19] Kodama N, Aoki Y, Hiraga H (1979). Electroencephalographic findings in children with moyamoya disease. Arch Neurol..

[20] Amzica F, Lopes da Silva FH, Niedermeyer E, Schomer DL, Lopes da Silva FH (2010). Cellular substrates of brain rhythms. Niedermeyer’s Electroencephalography: Basic Principles, Clinical Applications, and Related Fields.

[21] Evans BM (1976). Patterns of arousal in comatose patients. J Neurol Neurosurg Psychiatry..

[22] Foreman B, Claassen J (2012). Quantitative EEG for the detection of brain ischemia. Crit Care..

[23] Sharbrough FW, Messick JM, Sundt TM (1973). Correlation of continuous electroencephalograms with cerebral blood flow measurements during carotid endarterectomy. Stroke..

[24] Rogers JM, Bechara J, Middleton S (2019). Acute EEG patterns associated with transient ischemic attack. Clin EEG Neurosci..

[25] Cho A, Chae JH, Kim HM (2014). Electroencephalography in pediatric moyamoya disease: reappraisal of clinical value. Childs Nerv Syst..

[26] Kuroda S, Kamiyama H, Isobe M (1995). Cerebral hemodynamics and “re-build-up” phenomenon on electroencephalogram in children with moyamoya disease. Childs Nerv Syst..

[27] Kameyama M, Shirane R, Tsurumi Y (1986). Evaluation of cerebral blood flow and metabolism in childhood moyamoya disease: an investigation into “re-build-up” on EEG by positron CT. Childs Nerv Syst..

[28] Konishi T (1987). The standardization of hyperventilation on EEG recording in childhood II. The quantitative analysis of build-up. Brain Dev..

[29] Kuwabara Y, Ichiya Y, Sasaki M (1997). Response to hypercapnia in moyamoya disease. Cerebrovascular response to hypercapnia in pediatric and adult patients with moyamoya disease. Stroke..

[30] Lin Y, Yoshiko K, Negoro T (2000). Cerebral oxygenation state in childhood moyamoya disease: a near-infrared spectroscopy study. Pediatr Neurol..

[31] Kazumata K, Kuroda S, Houkin K (1996). Regional cerebral hemodynamics during re-build-up phenomenon in childhood moyamoya disease. An analysis using 99mTc-HMPAO SPECT. Childs Nerv Syst..

[32] Qiao F, Kuroda S, Kamada K (2003). Source localization of the re-build up phenomenon in pediatric moyamoya disease-a dipole distribution analysis using MEG and SPECT. Childs Nerv Syst..

[33] Touho H, Karasawa J, Shishido H (1990). Mechanism of the re-buildup phenomenon in moyamoya disease--analysis of local cerebral hemodynamics with intra-arterial digital subtraction angiography. Neurol Med Chir (Tokyo)..

[34] Kim DS, Ko TS, Ra YS (2006). Postoperative electroencephalogram for follow up of pediatric moyamoya disease. J Korean Med Sci..

[35] Boulos MI, Lena S, Han J (2014). Novel EEG pattern associated with impaired cerebrovascular reserve in moyamoya disease. Clin Neurophysiol..

[36] Garson SR, Monteith SJ, Smith SD (2018). Down syndrome associated moyamoya may worsen epilepsy control and can benefit from surgical revascularization. Epilepsy Behav Case Rep..

